# Lord Rosebery on the Increase of Insanity

**Published:** 1906-10-27

**Authors:** 


### Lord Rosebery on the Increase of Insanity.

The Edinburgh Lunacy Board have lately opened a village for lunatics at Bangour—a village presumably on the type of the one at Gheele, in Belgium. It is proposed that the patients shall work on the farm and in the shops just as they do in every asylum in the country; but possibly individual patients may have more liberty of action and more home comforts than in asylums of the older pattern. The cost of this village has been £300,000; and as it is intended for seven hundred or eight hundred patients, the cost works out at close upon £400 per bed, so that this plan of providing for the lunatic poor is far from being cheap; and it remains to be seen what advantages may accrue from it to compensate for the initial expense. As we write we have received information that the County Council of Essex are about to build another asylum at an estimated cost of £438,000. It would seem as if North and South vied with each other in the sums they expend on the housing of their pauper insane. And what is more wonderful is that these huge sums are voted by the people's representatives with scarcely a dissentient voice.

The Scottish Institution was formally opened by Lord Rosebery with a golden key, and a luncheon followed. We are glad to notice that Lord Rosebery declined to express any opinion on the proposal to establish lunatic hospitals for the preventive treatment of insanity—by which, we suppose, is meant institutions of some kind in which the disease might be treated in its very earliest stage—further than that, if tried at all, it should be tried on a limited scale, so as not to increase the present enormous expenditure. Although Lord Rosebery did not say it, it could not have escaped anyone possessing his rare acumen that insanity is one of the most terribly hereditary diseases known to medicine. Hence every so-called "cure" taking place in this generation will increase the amount of insanity in the next.

The enormous increase in the number of the registered insane during the last fifty years is a fact as undoubted as it is painful to contemplate; and it matters little to the ordinary ratepayer whether there be or be not an actual increase of insanity, inasmuch as whatever be the explanation of the fact that while the last half-century has not anything like doubled the general population, it has almost quadrupled the numbers of asylum lunatics; and the ratepayer has to pay for the vast majority of these patients. Lord Rosebery thinks—and we believe he is right—that Scotland and Ireland are in even a worse plight than England as regards the accumulation of asylum patients.

As regards the cost, his statements are appalling. The capital invested in asylums exceeds twenty-four millions, and three and a half millions are expended annually in the maintenance of the inmates. Nevertheless we can hardly accept his description of these asylums as sepulchres of living humanity and tombs of the intellectually dead. Such language is rather strong, but if it led his hearers to ponder over his facts it would be very ungracious to find fault with his methods. Plato or the idealist, Lord Rosebery went on to say, would advise the putting an end to this branch of the human race, and would check the marriages of those in whom there was any taint of insanity; and it was added that these would be excellent remedies if they were practicable. Of course, the first never has been, and never could be, even dreamt of; but why should Lord Rosebery say it is of no use to think of the suppression of such marriages as he refers to?

Almost ever since its inception this journal has pointed out to its readers that the reduction in the numbers of the insane did not lie in expensive and too often futile attempts at curing the disease, but in the development of methods to secure its prevention. It is probably under the mark to say that 50 per cent. of the present-day insanity is caused by heredity and drink; and Lord Rosebery believes that our only hope to reduce this tale of sorrow lies in the teaching of a higher and better system of life, and in the prevention of incessant restlessness, whether by motor-car, motor-bicycle, or railway carriage. This view may be correct if we have patience to look forward to some remote, indeed some very remote, future; but it is certain that not even Lord Rosebery's fascinating and seductive eloquence would prevent a single present-day lunatic from marrying if he had made up his mind so to do. His Lordship might as well sprinkle such individuals with rose-water as try by words to turn them from their matrimonial projects.

Quite lately one English County Council has seriously considered this burning question, and we hope that an attempt will be made to obtain legislative prohibition of the marriages of epileptics, habitual drunkards, and imbeciles. That such a step is needed no asylum physician of any experience will deny, nor, perhaps, would any member of Parliament privately deny it either.

